# Polyurethane-Based Electronic Packaging: The Characterization of Natural Aging over a Decade

**DOI:** 10.3390/mi16091061

**Published:** 2025-09-18

**Authors:** Xiaoqin Wei, Han Li, Rui Zhou, Changcheng Xie, Honglong Ning

**Affiliations:** 1 Southwest Technology and Engineering Research Institute, Chongqing 401329, China; weixiaoqin810913@163.com (X.W.); lihan3130@163.com (H.L.); xie5421367@126.com (C.X.); 2 Guangdong Provincial Key Laboratory of Energy and Information Polymer Materials, State Key Laboratory of Luminescent Materials and Devices, School of Materials Science and Engineering, South China University of Technology, Guangzhou 510640, China; 202421021262@mail.scut.edu.cn

**Keywords:** natural aging behavior, polyurethane, electronic packaging

## Abstract

Electronic devices with polyurethane electronic packaging have been stored in Chinese tropical marine atmosphere environments for 10 years. The long-term natural aging mechanism was studied by comparing the appearance inspection, molecular structure, elemental content, and chemical functional groups of the surface and interior of polyurethane electronic potting. The results indicated that, despite evident chemical aging and physical changes in the encapsulant material, it continued to effectively protect the internal electronic devices, maintaining their performance within an acceptable range. The interior polyurethane potting of electronic devices was white, but the surface turned yellow with noticeable color change. On the surface, the content of tolylene diisocyanate was greatly decreased. The peak heights of the internal carbamate groups located at 1708 cm^−1^ and 1529 cm^−1^ were significantly higher than those at the surface. In addition, the internal C element content for the carbamate group at 289.5 eV was higher than that of the surface. It can be inferred that, under ambient temperature and trace oxygen conditions, the urethane groups on the polyurethane electronic potting surface undergo aging reactions. These groups slowly oxidize into the quinoid structure of the chromophore, causing the surface to turn yellow. Despite this discoloration, the potting still protects electronic devices. Therefore, polyurethane electronic potting is ideal for the long-term sealed storage of electronic devices.

## 1. Introduction

In the production of electronic components, electronic potting is the most important step in finishing the electronic components. It can be said that electronic potting has become an indispensable and important insulating material in the electronics industry, improving reliability [1,2]. Electronic potting can improve the seismic resistance and waterproof performance of electronic components, greatly improving the reliability of electronic components. Without the use of electronic potting, electronic products are easily subject to erosion of the natural environment, leading to rapid line aging and the reduced service life of electronic products. Therefore, the protective role of electronic potting for electronic components is enormous [3].

Polyurethane electronic potting has excellent mechanical properties and electrical insulation properties and is widely used in electronic components. It prevents moisture, dust, and harmful gases from electronic circuit erosion, while also protecting electronic components from impact, improving anti-vibration abilities, anti-harsh environmental capabilities, and insulation properties of electronic devices. In addition, when used as part of a structure, polyurethane electronic potting strengthens the overall structure and allows for size reduction [4]. For long-term natural storage, the electronic circuit is in a non-working state. The failure mode is not the wear failure of the components. Polyurethane electronic potting is chemically and physically aged due to various environmental factors, resulting in the partial or complete loss of the physical, chemical, and mechanical properties of the polyurethane electronic potting, which eventually leads to the loss of the use value of the electronic circuit; therefore, the reliability change in electronic circuit depends on the reliability of the polyurethane electronic potting. It is important to examine the package reliability of electronic circuits after long-term storage.

At present, research on polyurethane potting mainly focused on the synthesis of new polyurethane composites, such as thermally sensitive shape memory polyurethanes [5]. Melamine-based polyether polyol (HMMM–PG) was synthesized to prepare polyurethane foams with high flame-retardant properties [6]. A graphene oxide (GO)-AZO dye composite was incorporated into PU-based coating to provide long-term durability against UV irradiation [7]. Moreover, the addition of semi-crystalline polyester diols to foam formulations shows exceptional tensile and elastic properties [8]. There is no accepted classification of climates for the degradation of materials and the topic of the degradation of synthetic materials is in its infancy [9]. Due to outdoor experimental degradation tests being rather time-consuming, there are few studies on the damage mechanism of polyurethane electronic potting during actual storage [10,11,12], and “faster” artificial degradation setups which mimic real outdoor conditions with higher stress factor intensities [13,14,15]. Hesham et al. [16] investigated the performance of polyurethane foam infill bridge deck panels (PU sandwich panels) after being exposed to various environmental conditions (a series of freeze–thaw, mid-high temperatures, and mid-high relative humidity cycles), which was conducted in a computer-controlled environmental chamber to duplicate seasonal effects in Midwestern states. However, there is a difference between accelerated test conditions and the actual storage environment, and there is always a fear of deviation from the chemical pathways of degradation for natural exposure because “relatively small variations in weathering performance can lead to significant variations in failure rates at a given time in service” [17]. Thus, the long-term natural aging mechanism of polyurethane has practical value, which will improve the production process and storage reliability, and also can be used for laboratory accelerated aging test method validation.

Moreover, the standards for material degradation are not uniform due to different climates, and extrapolations from short-term accelerated aging models or summaries of published natural exposure data remain insufficient to reliably predict long-term behavior [18]. Such methods cannot fully capture the complex, synergistic effects of multi-factor environmental exposure over extended periods, nor can they account for localized climatic variations and micro-environmental conditions. There is always a concern that accelerated testing may deviate from actual chemical degradation pathways, potentially leading to inaccurate lifetime predictions. Therefore, the use of actual ten-year storage samples is not merely preferable but necessary to provide unequivocal evidence of material performance.

In this paper, polyurethane electronic potting sealants for electronic components stored in a warehouse of tropical maritime climate conditions for 10 years were examined. The appearance, thermal decomposition, molecular structure, elemental content, and chemical functional groups on the surface and internally were compared using gray scale analysis, pyrolysis gas chromatography–mass spectrometry, X-ray photoelectron spectroscopy, and Fourier transform infrared spectroscopy. These analyses revealed the long-term natural aging mechanism of polyurethane electronic potting.

## 2. Materials and Methods

### 2.1. Materials

The polyurethane electronic potting provided by Xi’an Oriental Group Co., Ltd., Xi’an, China was used to conduct long-term natural storage tests. According to the material list provided by the manufacturer, the composition of polyurethane electronic potting material includes trimethylolpropane, toluene diisocyanate (TDI-80), and antioxidant D. The specific compound formulation and technological process were not disclosed because of commercial reasons. The materials were manufactured into electronic potting to improve the anti-vibration electronic components and anti-high overload impact.

### 2.2. Method

The water vapor transmission rate (WVTR) and oxygen transmission rate (OTR) of the packaging material were <0.02 g/m^2^/24 h and <0.05 cm^3^/m^2^/24 h/atm, respectively (test conditions: 38 °C, 90% RH). The pouches were heat sealed without vacuum or nitrogen purging, thereby encapsulating ambient air at the time of sealing. The high-barrier nature of the packaging material effectively shielded the samples from direct moisture ingress and significant humidity fluctuations. As a result, the aging process was predominantly influenced by long-term exposure to the trapped oxygen and trace moisture within the sealed environment, in combination with the ambient temperature.

### 2.3. Characterization

The color difference between the surface and interior of the polyurethane electronic potting material was visually evaluated in accordance with ASTM D1729 [19] under controlled diffuse illumination conditions.

In accordance with manufacturing and acceptance norms and product drawing requirements, sealing of electronic components, insulation resistance, and functional testing were carried out.

The information of molecular fragments was detected by AGILENT (6890N-5975C) pyrolysis gas chromatography–mass spectrometry from Agilent technologies (Santa Clara, CA, USA) and the molecular structure was deduced. Test method—Pyrolysis Program Setup: preheat to 150 °C (1 min) → purge for 0.5 min→ pyrolysis temperature 550 °C (0.5 min) → cleaning temperature 600 °C (0.5 min) → cool to 50 °C. Then, a HP-5MS fused sintered quartz capillary chromatographic column (inner diameter 0.32 mm) was used with high-purity helium gas as the carrier gas. The total carrier gas flow rate was 25 mL/min. The capillary carrier gas flow rate is 1.5 mL/min. EI mass spectrometry was employed, with an ion source temperature of 230 °C, a mass filter temperature of 150 °C, and an interface temperature of 250 °C.

XPS analysis was performed using the ESCALAB 250 analyzer (Thermo Inc., Waltham, MA, USA), equipped with an achromatic Al Ka X-ray source, and the excitation power was 150 W. The pressure during the analysis was 4.0 × 10^−8^ Pa. The survey spectra in the range of 0–1200 eV were recorded in 1 eV step for each sample. Atomic concentrations of each element were carried out by determining the relevant integral peak intensities. Fine narrow scans with step size 0.1 eV for C1s, O1s, N1s, and Si2p were recorded. The binding energies were corrected by referencing to the hydrocarbon component at 284.6 eV [20,21]. XPS provides information from the extreme outermost surface (~5–10 nm), revealing severe chemical changes such as oxidation and chain scission [20,21].

ATR-FTIR analysis was carried out using a Nicolet 470 Instrument (Nicolet Inc., Madison, WI, USA) in the range 420–3800 cm^−1^. ATR-FTIR spectra of the samples were taken at room temperature using the infrared radiation (IR) penetrates the surfaces of samples to approximately 1 μm. The average of three scans for each sample was taken for peak identification, with diamond as the ATR crystal. The sampling depths of different analytical methods are shown in Table 1.

## 3. Results and Discussion

### 3.1. Appearance Inspection and Electrical Properties Test of Electronic Components

**Figure 1:**
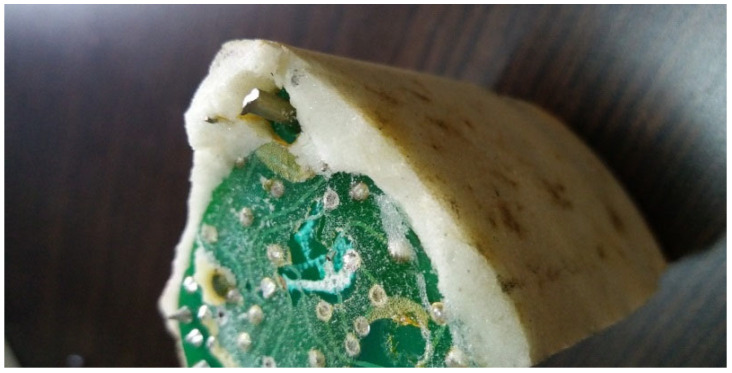
Appearance of polyurethane electronic potting sealants for electronic components stored in China tropical marine atmosphere environments for 10 years.

The appearance of polyurethane electronic potting sealants for electronic components stored in China’s tropical marine atmosphere environment for 10 years is shown in Figure 1. It can be seen that the surface of the polyurethane potting material turned yellow, while the interior remained white. Degradation of polymers typically begins at the outer surface and gradually penetrates into the bulk material [21]. In accordance with ASTM D1729 [22], the color difference between the surface and the interior was visually assessed under controlled illumination conditions. The results indicated a perceptible color difference equivalent to a slight but clearly visible contrast grade between the surface and the interior. 

Electronic components are located inside the packaging tube, hardly exposed to external temperature and humidity [23]. Therefore, polyurethane electronic potting is subject to the long-term impact of high temperature. Polyurethane electronic potting uses TDI as the raw material, in which the benzene ring is directly connected to the isocyanate group. The delocalized π-system on the benzene ring and the adjacent isocyanate group form a conjugate. Under the coupling action of heat and oxygen, the urethane bond is initiated to decompose and generate aromatic amines. Aromatic amine benzene nuclei are rearranged by oxidation, resulting in a quinone–imide structure which is a strong chromophore. Therefore, polyurethane electronic potting storage for 10 years tends to yellow and does not remain white long-term in water. Many papers on this subject showed that the yellowing of polyurethane electronic potting is a complex mechanism involving a series of reactions producing quinonoid-type structures [24,25].

The interior of polyurethane electronic potting is still white, which means that the degree of yellowing is not high, and then the oxidation has little effect on the physical properties of polyurethane. We conducted basic tests on the performance of electronic components. A total of five samples of electronic components were stored naturally in the tropical marine atmosphere for 10 years. The test results of their sealing performance, insulation resistance, and five key functional parameters are shown in Table 2. The standard deviation and coefficient of variation in these seven performance test results were calculated in accordance with GB/T 6379.2-2004 [26]. As can be seen from Table 2, the test results of the sealing performance, insulation resistance, and functional key parameters of the five electronic components did not exceed their respective technical index requirements. Moreover, from the calculation results of the coefficient of variation, the coefficients of variation in the seven performance parameters did not exceed 15%, with small volatility and good repeatability. This indicates that after long-term storage for 10 years, this type of electronic component, its sealing performance, insulation resistance, and functional parameters can still meet the technical index requirements.

**Figure 2:**
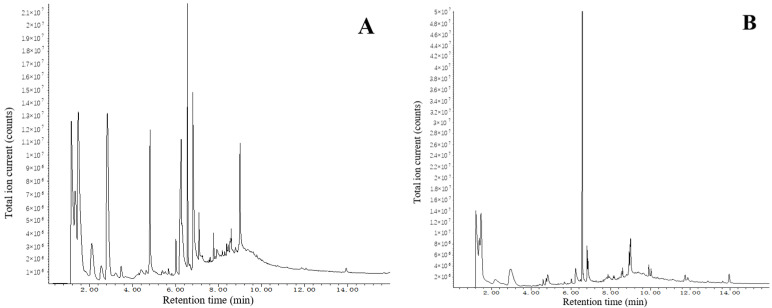
Py-GC/MS spectrum of polyurethane electronic potting stored for 10 years: (**A**) surface spectrum, (**B**) interior spectrum.

Py-GC/MS analysis was conducted to characterize the chemical composition of both the surface and interior of the polyurethane electronic potting material after 10 years of aging (Figure 2). The total ion chromatogram (TIC) peak area Normalization method was adopted to compare the changes in the mass fractions of the main components on the surface and inside, as shown in Table 3. The pyrogram revealed that fragments eluting between 2.2 and 6.5 min were primarily attributed to polyol-derived segments, while those between 6.5 and 7.0 min were indicative of isocyanate-related products.

The comparative analysis in Table 3 shows that the surface region exhibited a notable increase in the abundance of various polyol morphologies, along with elevated levels of phthalimide and 2-penten-1-ol. It is hypothesized that phthalimide may originate from the degradation of phthalate-based plasticizers or additives commonly used in polyurethane formulations, while 2-penten-1-ol likely results from oxidation and chain scission of polyol soft segments [25]. These changes are consistent with advanced surface degradation.

### 3.2. Elemental Content Analysis

**Figure 3:**
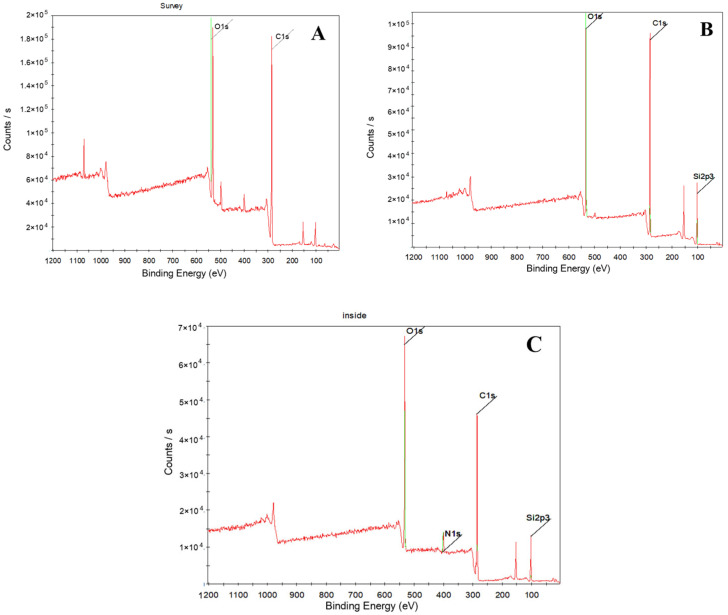
XPS full spectrum of polyurethane electronic potting: (**A**) Original sample; (**B**) Surface after 10 years of storage; (**C**) Interior after 10 years of storage.

**Figure 4:**
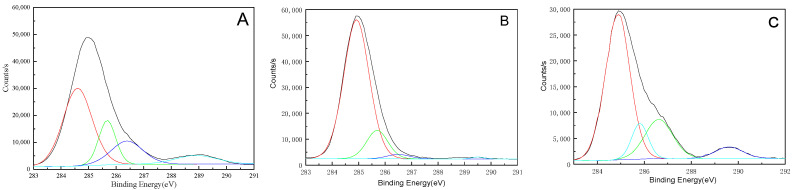
C1s peak fitting spectrogram of polyurethane electronic potting: (**A**) Original sample; (**B**) Surface after 10 years of storage; (**C**) Interior after 10 years of storage.

XPS spectra of the surface and the interior of polyurethane electronic potting stored for 10 years were displayed in Figure 3. It showed that the surface of polyurethane electronic potting compound mainly contains element carbon, oxygen, and silicon, but the interior of polyurethane electronic potting compound mainly contains elements such as carbon, oxygen, silicon, and nitrogen. A fine narrow scan of each element peak was carried out, and then Gaussian distribution was used to fit the C1s curve. The fitting principle was to make the fitting spectrum agree with the experimental spectrum, as shown in Figure 4. It showed that C1s peak on the surface and the interior of the polyurethane electronic potting are, respectively, superposed by four small peaks, indicating that there are four chemical environments for element carbon in the polyurethane electronic potting; that is, there are four chemical groups. Binding energy for four kinds of C1s fitting peaks were 284.9 eV, 285.7 eV 286.3 eV, and 289.5 eV, respectively. Based on the elemental standard chromatograms and their binding energy data in the compounds, four kinds of fitting peaks correspond to CH_2_-C_6_H_6_ (284.9 eV), C_6_H_6_-NH (285.7 eV), C–O–C (286.3 eV), and NHCOO (289.5 eV) [27,28]. According to the peak area of each element, the relative atomic content was calculated by using the sensitivity factor method, listed in Table 4. The charge correction was carried out based on the carbon–hydrogen pollution peak of 284.8 eV, and the Shirley mode was selected for background subtraction. Subsequently, the GL mixed peak type was chosen to fit the C1s curve. The fitting results are shown in Table 5.

The comparison of the relative atomic content obtained from C1s and N1s spectra (Table 4) reveals a noticeable decrease in the intensities of the NHCOO group (located at 289.5 eV) and the C–O–C group (located at 286.3 eV) on the surface. The nitrogen content in the interior is significantly higher than that on the surface, while the surface N1s signal falls below the detection limit, which may be attributed to possible siloxane contamination or carbon layer formation. Therefore, after 10 years of storage in a high-temperature environment, the polyurethane electronic potting material undergoes degradation primarily at the NHCOO groups, with more pronounced aging on the surface. This leads to reduced heat resistance and surface yellowing [28,29].

### 3.3. Chemical Functional Groups Analysis

**Figure 5:**
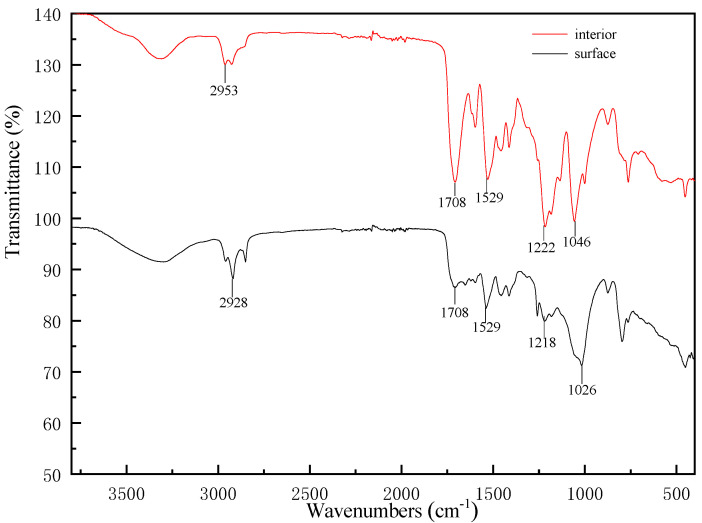
ATR-FTIR spectra of polyurethane electronic potting stored for 10 years (Spectrum vertically offset for clarity): interior and surface.

ATR-FTIR spectra of the surface and the interior of polyurethane electronic potting stored for 10 years were displayed in Figure 5. The peaks at 1026 cm^−1^ and 1046 cm^−1^ correspond to the stretching vibrations of aliphatic alcohol (C–OH), while those at 2953 cm^−1^ and 2928 cm^−1^ correspond to the stretching vibrations of aliphatic hydrocarbon (CH_2_). The peak at 1708 cm^−1^ is attributed to the stretching vibration of the carbonyl group in carbamate (C=O), 1218 cm^−1^ and 1222 cm^−1^ to the stretching vibration of carbamate (C–O–C), and 1529 cm^−1^ to the stretching vibration of carbamate (NHCOO) [30].

Due to the presence of a large amount of aliphatic alcohol (C–OH) in polyurethane electronic potting, the total amount does not change significantly even if the number of aliphatic alcohol group changes slightly, so the stretching vibration peak of aliphatic alcohol (C–OH) in 1026 cm^−1^ and 1046 cm^−1^ can be selected as the internal standard peak. The remaining peak height was compared with the internal standard peak height to study peak change characteristics of polyurethane electronic potting on the surface and interior, shown in Table 6.

Table 6 shows that the internal carbamate group located at 1708 cm^−1^ and 1529 cm^−1^ are much higher than surface carbamate group, indicating that aging degradation reaction occurs on the surface of polyurethane electronic potting stored for 10 years. The location of the degradation reaction at the carbamate group [31] results in the cross-linking density decreased and heat resistance reduced, which can powerfully support the conclusions of appearance inspection, Py-GC/MS, and XPS analysis.

### 3.4. Aging Mechanism Analysis

Multiple analytical techniques were used to compare the microstructural changes on the surface and in the interior of the polyurethane electronic potting material after 10 years of storage in a tropical marine atmosphere. The results indicate that long-term aging involves the combined effects of oxygen, temperature, and ultraviolet (UV) radiation.

Although the electronic components are sealed and hardly exposed to atmospheric humidity, the polyurethane surface remains exposed to atmospheric oxygen and sunlight. Under prolonged elevated temperature, the thermal energy facilitates the scission of the C–O bonds in the carbamate groups, initiating radical chain reactions. This leads to the formation of alkoxy and carbamoyl radicals, with the latter further decomposing into amino radicals and CO_2_. These reactions result in a noticeable decrease in carbonyl and carbamate content [31,32].

**Figure 6:**
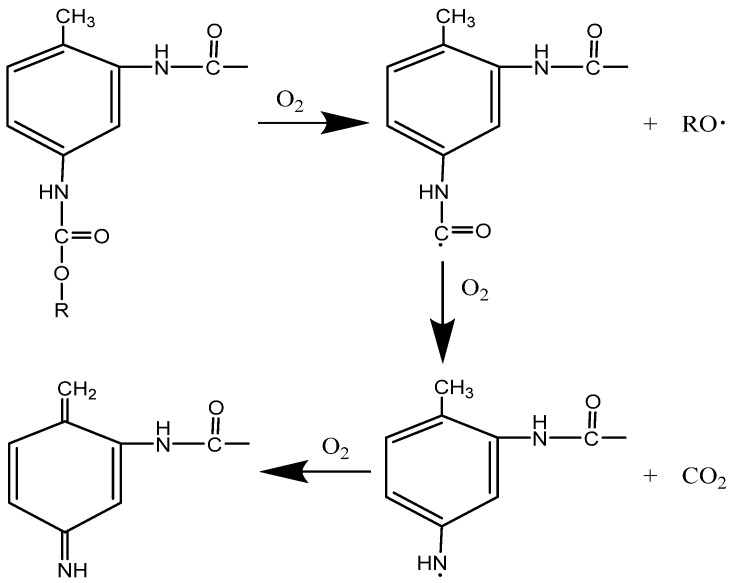
Long-term natural aging storage reaction of polyurethane electronic potting.

Simultaneously, UV radiation promotes surface cross-linking and oxidation processes. Amino radicals adjacent to aromatic rings undergo oxidation, forming large π-conjugated systems, such as quinone imine and other chromophores. These structures reduce molecular vibrational frequency, leading to a redshift in light absorption. When absorption occurs in the blue–violet region (∼400–450 nm) of the visible spectrum, yellow light is reflected, resulting in surface yellowing [33,34]. The decomposition of urethane bonds (hard segment disruption) is now explicitly linked to the observed decrease in the C1s component at 289.5 eV (XPS) and the reduction in IR absorption bands at approximately 1708 cm^−1^ (C=O stretch) and 1529 cm^−1^ (N–H bend coupled with C–N stretch). The subsequent formation of quinone-imine structures, which are responsible for yellowing, and the free radical chain aging reaction of polyurethane electronic packaging are shown in Figure 6. However, because the aging degree of thermal degradation for internal carbamate group is lower, it does not turn yellow by chemical action with oxygen, and the appearance is still white. At the same time, the macroscopic performance parameters of the electronic components are still within the specified range, indicating that the polyurethane electronic packaging still has the potting protection effect on electronic components after being stored for 10 years.

## 4. Conclusions

Polyurethane electronic packaging used for sealed storage of electronic components is not affected by ultraviolet light or humidity, with ambient temperature being the only influencing factor. Its weather resistance is good, and although surface yellowing occurs, the price is low and it is convenient to source, making it very suitable for the long-term sealed storage of electronic components. It should be noted that this study primarily focused on the chemical and morphological changes of aged polyurethane. Future work will include a systematic evaluation of mechanical properties—such as hardness, elastic modulus, and adhesion strength to comprehensively correlate chemical degradation with mechanical performance evolution

## Figures and Tables

**Table 1:** Sampling depth table of different information detection methods.

	Detection Site	Information Depth Range		
		XPS	ATR-FTIR	Py-GC/MS
Surface sampling depth	Outermost	Outside to the near surface atomic layer: 5–10 nm	0.5–3 μm	Outer surface sample
Inner sampling depth	Near-component inner	The inner surface; depth: 5–10 nm	0.5–3 μm	Inner sample

**Table 2:** Key performance test results of electronic components before and after aging.

Parameter	Leak (Pa·m^3^/s)	IR (GΩ)	I_l (A)	T_d (s)	I_sb (mA)	V_a (V)	I_max (mA)
Requirement	1.0 × 10^−5^	≥0.045	≥0.5	0.4~1 s	≤50	≤0.8	≤250
Sample 1 (Original)	8.1 × 10^−7^	99 GΩ	0.7	0.66	30.7	0.46	148.3
Sample 2 (Original)	8.6 × 10^−7^	99 GΩ	0.7	0.68	30	0.45	146.9
Sample 3 (Original)	7.3 × 10^−7^	99 GΩ	0.7	0.68	29.2	0.44	146.9
Sample 4 (Original)	7.5 × 10^−7^	99 GΩ	0.8	0.65	30.1	0.47	148.4
Sample 5 (Original)	6.9 × 10^−7^	99 GΩ	0.7	0.65	30.9	0.44	147.5
Mean	7.68 × 10^−7^	99 GΩ	0.72	0.664	30.18	0.452	147.6
Std Dev (S)	6.01 × 10^−8^	0	0.04	0.0136	0.5980	0.0117	0.6512
CV	7.83%	0.00%	5.56%	2.04%	1.98%	2.58%	0.44%
Sample 1 (10 years)	3.8 × 10^−6^	99	0.7	0.64	29.8	0.43	177.5
Sample 2 (10 years)	3.9 × 10^−6^	99	0.7	0.68	25.4	0.47	159.2
Sample 3 (10 years)	4.7 × 10^−6^	99	0.6	0.67	27.9	0.40	145.6
Sample 4 (10 years)	4.2 × 10^−6^	99	0.8	0.59	24.3	0.41	128.2
Sample 5 (10 years)	3.7 × 10^−6^	99	0.6	0.60	30.9	0.45	135.7
Mean	4.1 × 10^−6^	99	0.68	0.636	27.66	0.432	149.24
Std Dev (S)	0.0000004	0	0.0748	0.0361	2.5113	0.0256	17.5334
CV	9.94%	0%	11%	5.68%	9.08%	5.93%	11.75%

Note: Parameter names are abbreviated in the table for clarity: Leak: Sealing property; IR: Insulation Resistance; I_l: Load current; T_d: Delay time; I_sb: Standby current; V_a: Closing voltage; I_max: Maximum operating current; Std Dev (S): standard deviation; CV: coefficient of variation.

**Table 3:** The mass fraction results of typical components of polyurethane potting compound.

Component	Toluene Diisocyanate	Phthalimide	2-Penten-1-ol
Surface	0.4%	27.5%	0.5%
Interior	1.1%	20.2%	0.1%

**Table 4:** Analysis results of relative atomic content of elements in polyurethane potting compound.

Aging Time	Element	C	N	O	Si
	Binding Energy/eV	285	400.4	532	102.2
0 year	Ontology/%	51.08	3.45	26.52	18.95
10 years	Interior/%	58.87	3.51	22.33	15.29
10 years	Surface/%	56.73	0	20.23	23.04

**Table 5:** The peak division fitting (GL mixed type) result of polyurethane potting compound C1s.

Aging Time	Binding Energy/eV	284.9	285.7	286.3	289.5
	Belonging Group	CH_2_-C_6_H_6_	C_6_H_6_-NH	O-CH_2_(CH_2_)_2_CH_2_-O	HN-COO
0 year	Ontology/%	56.87	16.31	18.07	8.75
10 years	Interior/%	65.04	10.31	19.16	5.49
10 years	Surface/%	81.00	12.06	5.76	1.18

**Table 6:** The ratio of peak areas of the characteristic peaks of polyurethane potting compound in FTIR.

Position	1046 cm^−1^	1708 cm^−1^	1529 cm^−1^	1222 cm^−1^	2953 cm^−1^
	C–OH	C=O	NHCOO	C–O–C	CH_2_
Interior	1028	1352	591	444	100
Surface	1067	220	335	517	100

## Data Availability

The original contributions presented in this study are included in the article. Further inquiries can be directed to the corresponding author.
